# Isolation of Extracellular Vesicles from Agri-Food Wastes: A Novel Perspective in the Valorization of Agri-Food Wastes and By-Products

**DOI:** 10.3390/foods13101492

**Published:** 2024-05-11

**Authors:** Raffaella Latella, Eleonora Calzoni, Lorena Urbanelli, Giada Cerrotti, Serena Porcellati, Carla Emiliani, Sandra Buratta, Brunella Tancini

**Affiliations:** 1 Department of Chemistry, Biology and Biotechnology, University of Perugia, 06100 Perugia, Italy; raffaella.latella@dottorandi.unipg.it (R.L.); eleonora.calzoni@unipg.it (E.C.); lorena.urbanelli@unipg.it (L.U.); giada.cerrotti@dottorandi.unipg.it (G.C.); serena.porcellati@unipg.it (S.P.); carla.emiliani@unipg.it (C.E.); brunella.tancini@unipg.it (B.T.); 2 Centro di Eccellenza sui Materiali Innovativi Nanostrutturati (CEMIN), University of Perugia, Via del Giochetto, 06123 Perugia, Italy

**Keywords:** agrifood, wastes, by-products, nutraceuticals, extracellular vesicles, bioactive compounds

## Abstract

Agri-food wastes generated by industrial food processing are valorized through the extraction of biomolecules to obtain value-added products useful for various industrial applications. In the present review, we describe the valuable by-products and bioactive molecules that can be obtained from agricultural wastes and propose extracellular vesicles (EVs) as innovative nutraceutical and therapeutic compounds that could be derived from agriculture residues. To support this idea, we described the general features and roles of EVs and focused on plant-derived extracellular vesicles (PDEVs) that are considered natural carriers of bioactive molecules and are involved in intercellular communication between diverse kingdoms of life. Consistently, PDEVs exert beneficial effects (anti-inflammatory, anti-tumor, and immune-modulatory) on mammalian cells. Although this research field is currently in its infancy, in the near future, the isolation of EVs and their use as nutraceutical tools could represent a new and innovative way to valorize waste from the agri-food industry in an ecofriendly way.

## 1. Introduction

The growing demand for food production and the associated processing industry due to the rapid increase in the world population represents one of the most current issues. The consequence of this is the generation of significant amounts of agri-food wastes and by-products whose management has a significant impact on the environment, economy, and society [1].

Waste from the agri-food industry consists of a variety of matrices with different compositions and could represent a resource of bioactive molecules. In the last few years, numerous studies have focused on the valorization of this waste by extracting biomolecules to obtain value-added products for applications in various industrial areas, including pharmaceuticals, cosmeceuticals, and the food industry [1]. Even if the extraction of bioactive compounds from agri-food waste has positive implications for the economy and society, several problems have to be solved such as the use of organic solvents for biomolecule extraction [2] and the preservation of their biological activities during processing and storage [3].

In this review, we analyze the possibility to use agri-food wastes as a source of extracellular vesicles (EVs), a heterogeneous group of membrane-surrounded nanostructures that carry functional and bioactive compounds [4,5]. EVs are released in extracellular environments from almost all types of cells and are involved in cell-to-cell communication between diverse kingdoms of life [6]. In particular, EVs derived from various plant species (PDEVs), i.e., edible plants/fruits, have been characterized and proposed as new nutraceutical and therapeutic tools [7]. PDEVs share some biophysical and biochemical features with EVs released by mammalian cells (M-EVs) [6]. PDEVs are natural carriers of bioactive molecules and exert beneficial effects on mammalian systems, such as anti-inflammatory, anti-tumor, and immune-modulatory [6]. PDEVs are also considered efficient therapeutic nanovectors for delivering poorly soluble molecules or therapeutic agents to target cells [8]. In fact, several modification techniques could be used to encapsulate in PDEV drugs or bioactive molecules or to load functional ligands on their surface, thus increasing the drug delivery efficiency [8]. So, PDEVs have been proposed as new nutraceutical and therapeutical tools as they overcome some limitations of M-EVs such as low yield, production cost, toxicity, and immunogenicity [6]. Since the presence of EVs in foods (i.e., milk and dairy products, edible plants, plant-based products, and fermented foods) has been well established [8], it is reasonable to hypothesize the presence of EVs in agri-food wastes and suggest that such wastes may become a valuable source of EVs, strengthening the concept of waste valorization. In this regard, the presence of nanovesicles in the olive vegetation water, a waste produced by the olive oil industry, has been recently demonstrated, [9]. It is noteworthy to mention that the extraction of bioactive PDEVs does not involve the use of organic solvents and/or polluting substances commonly used for the extraction of organic compounds. In fact, the current methods used to isolate PDEVs, such as ultracentrifugation and size exclusion chromatography, are cost-effective and ecofriendly [6]. In addition, the bioactive molecules ‘naturally’ packaged in PDEVs could allow for obtaining different types of PDEVs with different therapeutic effects.

## 2. Valorization of Agri-Food Wastes and By-Products

### 2.1. Management of Agri-Food Waste and By-Products

Agricultural and food waste management is becoming a global concern. The rapid growth of the global human population is the main cause of the significant amount of agri-food waste and by-products generated annually worldwide [1]. These wastes are produced along the entire agri-food supply chain (production, processing, and consumption), mainly by household and food processing industries, and accumulate with deleterious effects on the environment. The management of bio-waste also implies a high economic cost for disposal. FAO [10] has estimated that around 14% of the food in the world, referring only to the edible parts along the food chain, is lost or wasted between harvest and the retail level pre-treatment, causing significant environmental problems. Landfill waste promotes the uncontrolled growth of microorganisms that cause bacterial contamination of groundwater and the production of toxic gases that contribute to air pollution. In addition, increased greenhouse gas emissions have devastating effects on climate by contributing to global warming [11]. On the other hand, the incineration of agri-food waste causes the release into the atmosphere of ashes, fumes, and dioxin with serious consequences for the health of living organisms and, more generally, of the environment. Other conventional methods used to dispose of bio-wastes are their use as animal feed or the spread of the soil, or “composting”, eliminating the need for biofertilizers [12].

Although the reduction in agri-food waste and by-products is considered the first option, in the last few decades, the valorization of bio-wastes through their reuse and recycling to obtain bioenergy and high-value bio-products has been considered. Agri-food waste, in fact, is made up of low-cost renewable material rich in nutritional and functional compounds that can be used for various green chemical or biotechnological processes solving many economic, environmental, and social problems. Agri-food waste and by-products could be used as a substrate for the biotechnological production of biofuels, or they could be employed as feedstock for direct extraction or production through microbial fermentation of nutritional and functional components such as proteins, enzymes, antioxidants, vitamins, fine chemicals, biopolymers, and many other bioactive compounds of commercial interest or even for the bioconversion into biodegradable plastics [1].

This approach is in line with the circular economy concept whereby waste and by-products of industrial activities are the starting material for producing high-value products through sustainable processes from an economic and environmental point of view. Undoubtedly, a sustainable valorization of agri-food waste and by-products through their reuse for the production of bioenergy and value-added products can effectively reduce the amount of waste to be disposed of and help to relieve environmental stress and energy consumption.

### 2.2. Wastes from Agriculture and Industrial Food Production

Wastes from the agri-food industry consist of a variety of matrices with different compositions. During the processing of vegetables and fruits for packaging, the disposal of plant components such as leaves, stems, roots, and tubers is common [1]. The processing of tomatoes, as well as the production of fruit juices and olive oil, yields substantial quantities of residues, predominantly composed of husks, peels, seeds, pomace, and pulp [13]. Concurrently, the dairy industry generates wastewater and by-products, notably whey, while meat processing results in the production of animal carcasses [1]. Moreover, a substantial part of fruits and vegetables that do not meet quality requirements are discarded after harvest or before reaching the consumer [1]. However, due to the high content of molecules with beneficial effects on human health, bio-waste is a natural resource of functional compounds, as well as feed for livestock and for the production of bioenergy [14].

The beverage industry is estimated to produce 26% of the entire food chain waste [1]. Fruit pomace is highly perishable, and its accumulation creates severe technical and environmental problems. However, in many cases, fruit pomace contains much higher amounts of bioactive compounds than fruit juice itself and can effectively represent a natural source of a wide range of high-value-added products [15,16]. Among fruits, citrus fruits (especially orange), apples, and grapes are the most produced in the world, thus creating significant amounts of bio-waste along the entire food chain. About half of the citrus fruits produced are used in industrial processing for the production of juices, jams, and marmalades with the generation of about 50–70% of waste with respect to the wet weight of the processed fruit. This waste is used as animal feed or discarded without further treatment, thereby causing severe environmental problems [15]. However, more recently, processing citrus waste has been considered a valuable source of fiber-rich components for the food industry, as well as essential oil and phytochemicals [17]. By-products from citrus fruits include pectin, polyphenols, sugars, carotenoids, vitamins such as vitamin C and vitamin B complex, and lipids such as linolenic, oleic, palmitic, and stearic acids [18,19,20]. Apple is one of the most used fruits in the world. Apples are mostly eaten raw or used to produce juice, cider, jam, and vinegar. The pomace derived from the industrial processing of apples is traditionally used as animal feed and fertilizer [15]. However, apple pomace is also an important source of pectin and bioactive compounds, such as vitamins, phenolic acids, and flavonoids, with recognized antioxidant and anti-inflammatory capacity, and anthocyanins, which have anticancer and anti-inflammatory effects and play a role in the prevention of cardiovascular diseases [15,21,22]. Particularly, apple peel has been reported to contain about 80% phenolic content and 5–6 fold antioxidant potential, compared to apple pulp [23]. Grapes are mainly used for the wine-making process which is accompanied by the generation of considerable amounts of waste made up of stalks and pomace (a mixture of grape seeds, skin, and pulp). Grape pomace is used as a soil conditioner as a source of energy [24] and as animal feed [25]. These wastes are rich in many different phenolic compounds with proven beneficial effects such as cardio- and neuro-protection, anticancer, anti-inflammatory and anti-pathogens activity [26,27,28]. Grape pomace also contains resveratrol and flavonoids which have nutraceutical properties, while grape seeds are a rich source of polyunsaturated fatty acids and vitamin E, a lipid-soluble antioxidant [26,29].

Another agri-food industry that generates a huge volume of waste is the olive industry (mostly concentrated in the Mediterranean area), as olive vegetation water or olive pomace represents about 40% of the entire mass of processed olives [30,31]. Defatted olive pomace can be used as biofuel through lipid transesterification. However, defatted olive pomace retains important nutritional and functional compounds, such as proteins and phenolic compounds, which account for 5–10% of the feedstock [30,32,33,34]. The effective valorization of olive processing waste through the extraction of these valuable compounds could represent a useful alternative to the disposal of waste from the olive oil industry allowing a reduction in environmental contamination [30].

Tomato is one of the most used vegetables in the food industry (mostly to produce soups, ketchup, and juice), generating a significant amount of waste. It has been estimated that waste generated by tomato industrial processing ranges from 5 to 19% of the incoming mass [30]. Residues and by-products from tomato processing include discards, peels, seeds, and pulp that have a nutritional value similar to the pre-processed material [30]. Currently, tomato waste is mostly used for animal feed or as fertilizer [26]; however, new extraction techniques have been taken into account to obtain valuable compounds with the advantage of improving the management of tomato food chain waste [26]. In fact, tomato residues are rich in carotenoids and polyunsaturated fatty acids with recognized antioxidant and health-promoting properties [35,36]. Moreover, tomato seeds contain pectin [37] and high-quality proteins [26,38].

In addition to fruit and vegetable processing, dairy and meat industries also contribute significantly to the food chain wastes. Most of the dairy industry waste comes from the production of cheese and consists of whey, which is the aqueous fraction left over from the milk after casein coagulation, and wastewater. These bio-wastes represent a real threat to the ecosystems and their disposal poses serious environmental problems due to their high nutritional value, which can cause excessive growth of microorganisms and aquatic plants [39]. Dairy industry waste is rich in proteins and carbohydrates with important nutritional and functional properties and can be effectively used as a source for the preparation of various food derivatives [15]. Moreover, bioactive peptides with antioxidant and anti-hypertensive properties can be obtained via enzymatic hydrolysis of whey proteins [40,41]. More recently, the transformation of dairy industry waste and by-products into value-added compounds through biotechnological processes has been considered. This approach proved that dairy waste can be a good source to produce many different materials such as bioplastics, biofuels, organic acids, and biosurfactants [11]. Industrial meat processing also significantly affects the environment contributing to climate change. In fact, despite ethical and health concerns about excessive meat consumption, intensive livestock farming as a source of protein for the human diet is growing as the global human population grows. Industrial meat processing generates a significant amount of waste mainly consisting of skin, bones, cartilage, blood, viscera, etc., the disposal of which is particularly complex and highly regulated due to the risk of spreading encephalopathies [42]. Most of these by-products are rich in nutrients such as proteins, vitamins, and minerals and can be reused as ingredients for human foods and animal feed or for chemical and biomedical applications [43]. In particular, meat by-products are rich in proteins and can be exploited as nutraceutical ingredients for the preparation of innovative foods as well as fertilizers and pharmaceutical products [44]. Their use as building blocks for developing new sustainable technologies for biodegradable bioplastics, water purification, and renewable energy has also been considered [42].

In addition to these most representative examples of wastes and by-products generated by the agri-food industry, there are many other food supply chains that contribute to the huge volume of waste produced annually by food processing and which could be considered for the presence of important nutraceutical and bioactive compounds. Examples of these may be germ and bran from cereals, seeds from fruits and vegetables, edible seaweed harvest, and fish and shellfish wastes [15]. Table 1 summarizes the main types of agri-food waste and their management.

### 2.3. Bioactive Compounds from Agri-Food Waste and By-Products

Numerous studies have recently focused on the sustainable valorization of agri-food waste and by-products to obtain value-added products for applications in various industrial sectors, including pharmaceuticals, cosmeceuticals, and food. These studies have shown that agri-food waste contains compounds with a wide range of biological functions such as antioxidant, anti-inflammatory, anticancer, or antibacterial properties that could provide beneficial effects on human health [45,46].

Phytochemicals and nutraceuticals obtainable from agri-food waste and by-products include carbohydrates, pectin, proteins, peptides, lipids, fatty acids, phenolic acids and polyphenols, flavonoids, carotenoids, vitamins, anthocyanins, pigments, and more. However, pectin, phenolic compounds, anthocyanins, and natural pigments (i.e., carotenoids) are currently bioactive compounds mostly extracted from vegetable and fruit by-products [47]. 

#### 2.3.1. Bioactive Compounds from Waste of Plant Origin

Pectin is mainly produced from apple pomace, citrus peels, and, to a lesser extent, sugar beet [48]. Pectin is a complex polysaccharide composed mostly of galacturonic acid and rhamnose with side branches of galactose or arabinose and is found in the primary wall of non-woody plant cells, where it serves to cement the space between the cells, keeping them together and giving consistency to the fruit. As ripening progresses, the pectin is enzymatically hydrolyzed, and the fruit loses consistency. Pectin has gelling, thickening, and stabilizing properties due to the easy association of pectin chains in water leading to the formation of a three-dimensional network stabilized by the formation of inter and intra-molecular hydrogen bonds, hydrophobic interactions, and/or ionic bonds. The high hydrophilicity of pectin contributes to its biodegradability, biocompatibility, and low toxicity [49]. For this reason, it is routinely used as a food supplement (E440) for the preparation of sauces, jams, sausages, canned foods, and bakery. However, pectin also has many health-promoting properties and finds wide biomedical, pharmaceutical, and cosmetic applications [48]. The beneficial effects of pectin as a nutritional supplement include a reduction in the cholesterol level in plasma and liver, a reduction in glucose intolerance in diabetic patients, and a lowering of blood pressure [49,50]. In addition, in vitro and in vivo studies indicated that pectin has immunomodulatory and anti-inflammatory activities and intestinal microflora-regulating properties [48,49], and it can also be effective as an antidote against various harmful agents such as heavy metals, long-life radionuclides, and free radicals [51]. In addition, pectin has also been shown to have anticancer properties, and recent studies have reported that pectin and its derivatives from various sources have anti-proliferative effects on various cancer cell lines [49]. In the pharmaceutical industry, due to its biocompatibility and biodegradability, pectin is used for various purposes including bioactive and drug delivery, biodegradable film and coating preparation, nano- and micro-encapsulation, wound dressings, and tissue engineering scaffolds [52]. Moreover, pectin can be combined with other organic or inorganic compounds or polymers to modify its physicochemical characteristics and functionalities and obtain composite materials with specific properties for targeted applications [53]. Pectin also finds wide applications in the cosmetic industry for the preparation of skin care products such as gels and facial masks [51]. Currently, worldwide researchers are investigating the properties of pectin, always discovering new possible applications to extend its use in various industrial sectors.

Agri-food waste and by-products could also be a good source of phenolic compounds. Phenolic compounds constitute one of the most numerous classes of bioactive molecules widely distributed in edible plants and are considered among the main important phytochemicals for both their potential application as food preservatives and their beneficial effects on human health. These compounds represent a large family of plant secondary metabolites whose structure presents an aromatic ring with at least one hydroxyl group and can vary from simple molecules to highly complex polymers [54]. Based on their chemical structure, polyphenols are classified into five main groups, namely, phenolic acids, flavonoids, stilbenes, tannins, and coumarins [55]. These compounds play an important role in plant growth and reproduction, providing effective protection against pathogens and parasites. In addition, polyphenols determine the sensory characteristics of fruits and vegetables by contributing to color, taste, and flavor, as well as astringent and bitter tastes [56]. For their antioxidant and antibacterial activities, in recent years, plant-derived phenolic compounds have been utilized in the food industry to improve the shelf-life and quality of foodstuffs during storage [56,57,58]. In addition, evidence from epidemiological studies revealed a clear correlation between the consumption of foods rich in phenolic compounds and a reduced risk of developing diseases such as chronic diseases and cancer [59,60,61]. Many food polyphenols are bioactive compounds with health-promoting and disease-prevention activities due to their antioxidant, antimicrobial, cardioprotective, anti-hypertensive, anti-inflammatory, and anticancer properties [46,62]. In particular, in vitro and in vivo studies have demonstrated that, compared to the edible part of the fruit, citrus peel contains a higher amount of total phenolic compounds with antioxidant, anti-inflammatory, and anticancer properties [19], and apple pomace is rich in phenolic compounds with radical scavenging activity, with the ability to inhibit protein glycation and anti-tumor activity [15]. The olive mill waste is also an important source of phenolic compounds with recognized antioxidant, antimicrobial, and nutraceutical activities [45,63,64]. In addition, phenolic compounds extracted from olive vegetation water can be used to improve the conservation and quality of food, such as meat and fruit [65,66], and as functional ingredients [67,68]. Polyphenols have also been used for industrial applications in the production of paints, paper, and cosmetics.

Anthocyanins, common flavonoids found in a wide variety of food industry wastes, are water-soluble natural pigments with blue, red, and purple pigmentation, depending on their structure and pH [69]. In particular, wastes and by-products from wine production and the juice industry can be used as good sources of anthocyanin pigments. For example, berry residues, apple peel, and grape pomace present a high content of these valuable natural colorants [69]. The use of anthocyanins as colorants in the food industry has recently been considered to replace synthetic food colors which have been banned due to their toxicity and carcinogenicity [56]. Anthocyanins also possess antioxidant and anti-inflammatory properties [70], which have been correlated with various positive effects on human health, including reduced risk of cardiovascular disease [71], cholesterol level regulation [72], and cognitive function improvements [73]. Therefore, their use as food coloring could provide enhanced benefits.

Agri-food waste and by-products are also rich in natural pigments (betalains, carotenoids, and chlorophylls), which are widely required by industries for food, pharmaceutical, and cosmeceutical applications due to their coloring and pharmacological properties [69]. Similarly to anthocyanins, there is a growing demand for these compounds to replace synthetic pigments; as a result, the need for environmentally sustainable technologies for their extraction is becoming more and more important. In this regard, betalains, which are water-soluble nitrogen pigments with red-purple/yellow-orange color, have been successfully extracted by ecofriendly methods from beetroot pomace [74] and peels of red dragon fruits [75] and prickly pears [76]. Carotenoids are lipophilic pigments responsible for the red, yellow, or orange color of many fruits and vegetables, enriched in tomatoes, carrots, spinach, mandarins, maize, and watermelons. It is noteworthy to mention that the nonedible parts of plants, such as peels and leaves, are the richest in carotenoids and are currently used to extract these valuable pigments for their use as a food additive [77]. Chlorophylls, oil-soluble amphiphilic pigments, are widely distributed in plants and algae and could be extracted from many types of agri-food waste to be used both as coloring pigments and as nutraceutical agents. In particular, spinach, lettuce, and broccoli, which all generate a great amount of waste, are the main vegetable sources of chlorophyll [77]. Another source of chlorophylls is microalgae residues which contain very high concentrations of these pigments [77]. Pigments are mainly used by food industries as color intensifiers and additives. However, all these types of natural pigments have been reported to exhibit numerous beneficial effects on human health, including antioxidant, anti-inflammatory, anticancer, antimicrobial, cardio-, and neuro-protective activities, and therefore, are also considered for their use as functional ingredients in food products [69,77].

#### 2.3.2. Bioactive Compounds from Waste of Animal Origin

Dairy and meat processing waste and by-products represent a rich source of proteins. Whey is commonly used to produce whey protein concentrates which, due to their excellent nutritional value [11], are added to foods for high-protein diets. Whey proteins are also used as emulsifiers and gelling agents for the preparation of baked and confectionery products, yogurt, and ice cream [42]. α-Lactalbumin, an abundant whey protein involved in the synthesis of milk lactose, has been reported to possess anti-hypertensive, antioxidant, anti-obesity, and anticancer properties and can be used in biomedical applications [78]. Meat processing by-products such as skin, cartilage, and bones are commonly used to extract collagen, both in native or partially hydrolyzed form. Collagen has a low nutritional value and is not considered for nutritional applications. However, this protein and its derivatives are extensively used for various applications in the food, pharmaceuticals, and cosmeceutical industries. Gelatin, the hydrolyzed form of collagen, is used in the food industry as an emulsifying and gelling agent [79], whereas hydrolyzed type I and II collagen are used as dietary supplements to promote collagen production to counteract aging and drying of the skin [80]. Collagen peptides have been reported to have bioactive properties showing beneficial effects on joint health and osteoarthritis [81]. Collagen also finds many applications in cosmetics [82] and exhibits properties suitable for regenerative medicine applications [83]. Meat processing waste is also used to produce processed animal proteins, which are utilized for the preparation of feed intended exclusively for pets and fish, while they are banned for feed for farm animals due to the risk of the encephalopathies spreading [84]. Another interesting use of meat by-products is the production of small bioactive peptides by controlled enzymatic digestion. These peptides have been shown to exhibit different biological activities, including anti-hypertensive, antioxidant, antithrombotic, and antimicrobial effects [84]. Blood, an abundant by-product of meat processing, is rich in proteins with high nutritional and functional properties that can be exploited for various technological applications such as gelling, foaming, and emulsifying agents, while albumin, the most abundant plasma protein, is used in medical applications as a stabilizer in vaccines and in antibiotic sensitivity tests [85].

Table 2 summarizes the main classes of bioactive compounds found in agri-food waste and their related properties and biological activities.

### 2.4. Limitations and Possible Solutions in the Re-Use of Agri-Food Waste and By-Products

Although the extraction of bioactive compounds from agri-food waste and by-products to obtain products with high added value can represent a useful strategy, there are still several problems to be solved for their effective application in the productive sectors. In most cases, pre-treatments of agri-food waste and by-products are necessary to optimize their handling and storage. Furthermore, extraction processes are expensive, energy and time consuming, and the yield is low and must be optimized in order to be applied on an industrial scale [47]. In addition, the main challenge for the utilization of bioactive compounds from agri-food waste and by-products comes from their high instability and susceptibility to different factors during processing and storage that can affect their bioavailability and bioactivity. Indeed, most of them are prone to degradation due to their sensitivity to heat, light, pH, and oxygen which can cause chemical structural changes and biological inactivation [3]. In addition, many phytochemicals have low solubility which makes their incorporation into some foods or preparations difficult, leading to low bioavailability. Another challenge is that most of them are degraded in the gastrointestinal tract with consequent reduction in bioactivity. To overcome many of these problems, recent studies have proposed the application of novel nanotechnological techniques (i.e., micro- and nano-encapsulation) to improve the chemical stability, solubility, and bioavailability of natural bioactive substances and allow their controlled delivery and release [45,55]. Encapsulation consists of trapping the bioactive compounds inside nanoparticles (maximum particle size of 500 nm) made up of suitable edible biopolymers, which provide protection to the phytochemicals enhancing their shelf-life and improving their bioactivity and effectiveness. Micro- and nano-encapsulation are versatile methods that can meet different needs and requirements as the properties and functionality of the nanoparticles can be tuned for different applications by changing their physicochemical characteristics, such as composition, size and shape, and method of preparation [1]. This nanotechnological approach can contribute to the development of innovative products and new applications especially for the food and pharmaceutical industries, while improving the management of agri-food waste and by-products.

## 3. Plant-Derived Extracellular Vesicles as Natural Carriers of Bioactive Molecules

### 3.1. Extracellular Vesicles

Extracellular vesicles (EVs) are a heterogeneous group of membrane-surrounded structures, which are released in an extracellular environment from almost all types of cells; they circulate in body fluids (i.e., blood, milk, urine, saliva, and ascites) and are involved in cell-to-cell communication [4,5]. Recent growing interest in EVs is due to their peculiar properties. In fact, EVs isolated from biological fluids represent potential biomarkers for the diagnosis and follow-up of various pathologies, as their biochemical cargo reflects, at least in part, the type and pathophysiological conditions of the releasing cells [86]. Furthermore, the ability of EVs to transfer biological information to specific target cells makes them good candidates for therapeutics and/or drug delivery, as they are able to overcome natural barriers (i.e., blood–brain barrier) and are stable in biological fluids and gastrointestinal tract [87]. However, the use of EVs for drug delivery is still limited as it is necessary to develop suitable and reproducible methods for their isolation, drug loading, and in vivo biodistribution improvement [87].

EVs are classified into different classes based on their biogenesis mechanisms, size, and stimuli, that induce their release by parental cells. In mammals, the most well-characterized EV classes are as follows: exosomes (30–100 nm) derived from the endosomal system; microvesicles (100–1000 nm), originating from membrane shedding; apoptotic bodies (1000–5000 nm) that are formed during the process of apoptosis [4]. Recently, additional classes of EVs have been discovered [88], such as exomers (30–50 nm) that are extracellular nanoparticles enriched in Argonaute 1–3 and amyloid precursor protein [89], autophagic EVs that are formed during autophagy [90], mitovesicles originating from mithocondrion [91] and oncosomes released by metastatic cancer cells [92]. These heterogeneous classes of vesicles individually or in synergy are involved in intercellular communication regulating several physiological and pathological processes.

EVs contain a set of conserved lipids and proteins. Regarding lipid composition, EVs are enriched in lipids usually associated with lipid rafts such as cholesterol, sphingolipids, and glycerophospholipids containing saturated fatty acids [93,94,95]. Unlike cellular membranes, EVs have a higher content of phosphatidylserine in the outer leaflet, which may facilitate their internalization by target cells, as well as lipids that provide stability in the extracellular environment [96]. EVs are also enriched with a collection of cytosolic and membrane proteins, e.g., annexin II and heat shock proteins, MHC class II complexes, integrins, and tetraspanins (CD9, CD63, CD81), which are commonly considered protein markers of EVs [97]. In addition to lipids and proteins, nucleic acids are also packaged in EVs. The presence of functional mRNA, i.e., which can be translated after transfer into another cell, was demonstrated for the first time in exosomes released by mast cells, and this supports the hypothesis that exosomes may represent a carrier for the transfer of mRNA, enabling to modulate the level of specific proteins in target cells [98]. In more recent studies, evidence has shown that EVs carry various types of RNAs, including miRNA, small interfering RNA, vault RNA, transfer RNA, long non-coding RNA, Y RNA, piwi-interacting RNA, and small nucleolar RNA. These RNAs may serve functional roles after their transfer into recipient cells [99]. RNAs are not the only nucleic acids present in EVs; the presence of mitochondrial DNA has been revealed in EVs released by glioblastoma cells and astrocytes [100], and the presence of dsDNA has been demonstrated in EVs released by tumor cells [92].

### 3.2. Plant-Derived EVs

**Figure 1:**
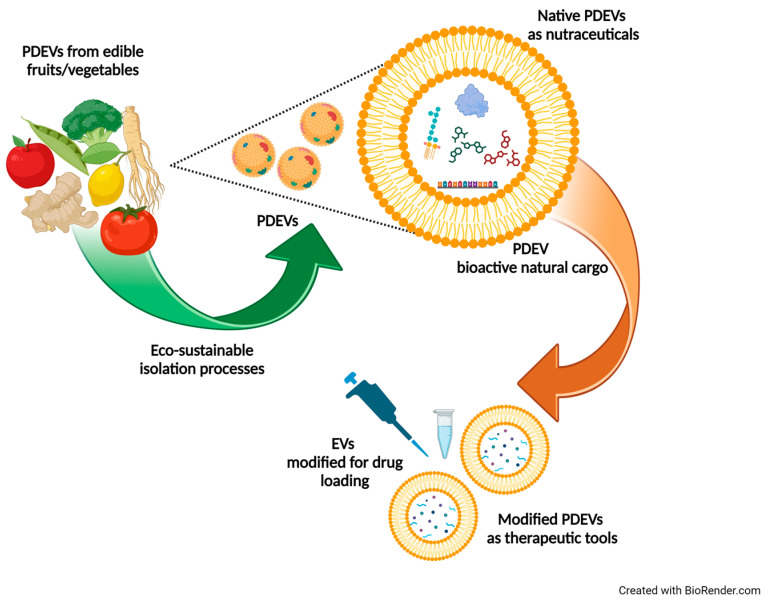
PDEVs, extracted sustainably from fruits and vegetables, can serve as natural nutraceuticals or can be tailored for therapeutic applications.

The majority of research in the EV field has been carried out in mammalian systems [101]. However, in recent years, an increasing number of studies have demonstrated that EVs can be isolated from different parts of plants including juice, pulp, roots, seeds, and dried plant materials [102]. Plant-derived EVs (PDEVs) are membrane-delimited nanostructures characterized by specific features in terms of morphology, size, and biochemical composition [102]. PDEVs are taken up by target cells and mediate cell-to-cell communication between diverse kingdoms of life. PDEVs contain bioactive molecules exerting antioxidant/anti-inflammatory effects [103]. Due to these properties, together with their stability in the gastrointestinal tract, they are considered a promising therapeutic tool (Figure 1) [8].

PDEVs share some features with mammalian EVs (M-EVs) but also have some characteristics that identify them. Like M-EVs, one pathway of PDEV biogenesis involves the endosomal complex. The vesicles destined for extracellular space are formed as intraluminal vesicles (ILVs) in the multivesicular body (MVB) and are released via fusion between the MVB and the plasma membrane. The formation of ILVs and MVBs requires the presence of the ESCRT complex. This works with a ubiquitin-dependent mechanism, binding ubiquitinated proteins and sorting them into the ILVs of MVBs [104]. ESCRT complex works in mammalian and plant cells likewise; the only difference concerns ESCRT-0 which is not present in the plant cells and is substituted by the TOM1 and TOM-like proteins that are responsible for ubiquitinated protein binding during MVB cargo sorting [105].

Other biogenesis pathways involve different cellular structures such as EXPO (exocyst-positive organelle), vacuoles, and autophagosomes. EXPO is a spherical organelle enveloped by a double membrane that fuses with the plasma membrane and produces another class of EVs [106]. The diameter of EXPO-derived EVs is between 200 and 500 nm [106]. Another way of biogenesis involves the vacuoles. The study of Cui and co-workers [107] demonstrated that ILVs are transferred inside the vacuoles through their fusion with MVBs. Then, vacuoles fuse with plasma membranes releasing vesicles extracellularly. This mechanism of biogenesis is activated after the infection of plant cells by bacteria [108]. The mechanism of PDEV biogenesis that involves autophagosomes is not clear yet [102].

After their biogenesis, PDEVs have to cross the cell wall (an essential structure of plant cells). However, the pore size of the cell wall is smaller than the average diameter of vesicles and it has been hypothesized that the enzymes packaged into PDEVs, already known for their involvement in cell wall remodeling, might create a transient destabilization in the cell wall structure, allowing vesicle passage through the cell wall and their subsequent release outside. Alternatively, it has been suggested that reversible stretching of plant cell walls determined by its natural plasticity or local breaks may help PDEVs to pass through it [104].

Analogously to the M-EVs, the biophysical characterization of PDEVs is provided by particle size distribution, zeta potential, and morphology. The zeta potential of PDEVs ranges from −70 mv to approximately neutral [109,110], while M-EVs’ zeta potential ranges from −10 mv to −50 mv [111]. Like the M-EVs, PDEVs have a cup-shaped morphology.

Biochemical characterization of PDEVs revealed that biomolecules carried by PDEVs are able to mediate metabolic and signaling pathways in the target cells, similarly to M-EVs [102]. Although some surface protein markers of M-EVs have been established, such as membrane transport proteins, or tetraspanins such as CD63, CD9, and CD81 [112]; no surface markers of PDEVs have currently been established [113]. The protein amount of PDEVs is lower than that of M-EVs, and most PDEV-associated proteins belong to the families of aquaporins, involved in the regulation of cell turgor; HSPs, related to biotic and abiotic stress responses and plant growth; metabolic enzymes; and annexins, implicated in intracellular vesicle transport and secretion [114]. Complete profiling of proteins carried by PDEVs is quite challenging since different plants require different sets of protein databases for matching [113].

An interesting difference between PDEVs and M-EVs is related to their lipid compositions. M-EVs are enriched in cholesterol and sphingomyelin, whereas PDEVs do not contain cholesterol and are enriched in phosphatidic acid and digalactosyl monoacylglycerol [115]. This peculiar lipid composition affects the stability of PDEVs and is crucial for interspecies and inter-kingdom communication and for their targeting of recipient cells [116].

Like M-EVs, the biochemical cargo of PDEVs is characterized also by the presence of nucleic acids, in particular DNA, mtDNA, mRNA, miRNA, and lncRNA [109,117]. miRNA are the most representative nucleic acids in PDEVs as shown in vesicles derived from ginger, grape, grapefruit, lemon, broccoli, and apple [102]. PDEV-associated RNAs are able to regulate gene expression and biological functions in target cells through cross-species interactions [118,119].

The metabolomic profile of PDEVs is highly variable because different plants produce a wide range of bioactive molecules involved in primary and secondary metabolism [102]. Some secondary metabolites packaged in PDEVs are flavonoids, chlorophylls, and curcuminoids, depending on the plant/fruit from which the vesicles are derived. A complete study of the metabolomic profile of EVs isolated from grapefruit was conducted by Stanly and co-workers [120]. They separate microvesicles (MVs, between 350 and 700 nm in diameter) and nanovesicles (NVs between 50 and 80 nm in diameter) from juice grapefruit using differential ultracentrifugation. Grapefruit-derived MVs contain sugars (i.e., monosaccharides, disaccharides, and their derivatives) and organic acids (i.e., quinic acid and oxalic acid). Notably, MVs also contain aucubin, an iridoid glycoside which has been shown to have a variety of pharmacological effects, including antimicrobial and anti-inflammatory effects [120,121]. Grapefruit-derived NVs have a different metabolic profile compared to MVs, as they are characterized by high amounts of organic acids, such as alpha-hydroxy acids (AHAs) (i.e., glycolic and citric acids). The amino acid content of grapefruit-MVs and grapefruit-NVs is mainly restricted to leucine/isoleucine and aspartic acid, the latter present only in grapefruit-MVs.

Several protocols already used to isolate EVs from mammalian cell culture media or biological fluids have been adapted to isolate PDEVs. Proper purification methods are crucial for the analysis of vesicles as they are often present within an overly complex matrix [113]. The widely used method is differential ultracentrifugation followed by density gradient centrifugation, as it is a simple and moderately time-consuming protocol [122]. Other methods used for the isolation are size-based isolation methods like ultrafiltration and size exclusion chromatography, which could be combined in order to obtain a population enriched with similar-sized PDEVs [123,124]. Each method has advantages and drawbacks; two or more methods can be combined to obtain purer samples enriched in a specific class of PDEVs.

Recent studies have demonstrated that PDEVs play multiple biological roles, including plant defense against pathogens, development, cell wall remodeling, reorganization of cell structure, crosstalk between plants and fungi, and plant immunity [102,104]. It is well documented that PDEVs have an active role against pathogens. It has been demonstrated that the proteome of EVs isolated from the apoplastic fluid of Arabidopsis thaliana leaves is enriched in proteins involved in biotic and abiotic stress response. In particular, it has been reported that the secretion of PDEVs is enhanced during bacterial infection by Pseudomonas syringae or in response to treatment with salicylic acid [125]. These findings suggest that PDEVs may represent an important component of plant immune response. The protein profile of PDEVs purified from tomato is also characterized by the presence of proteins known to be involved in plant–microbe interactions such as endochitinases, glucan-endo-1,3 beta glucosidase B precursors, and putative late blight resistance protein homologs [126]. PDEVs from sunflower seeds released in response to abiotic stress carry some signal peptides, which can transmit warning signals to nearby cells [127]. PDEVs have also been identified in the phloem of plants, a tissue that transports sugars to metabolically active tissues. The presence of PDEVs in a strategic tissue such as the phloem supports their role in intercellular communication in plants [104].

Sunflower-derived EVs carry proteins associated with carbohydrate metabolism. Many of these proteins are involved in the degradation and reorganization of the cell wall, such as glycosyl hydrolases, expansins, and arabinogalactan proteins [128]. Cell wall remodeling-related proteins have also been identified in Arabidopsis apoplastic vesicles [125]. Thus, these results indicate that PDEVs accumulate in the apoplast and move towards extracellular space crossing the cell wall.

### 3.3. Functional Properties of PDEVs Isolated from Different Plant Sources

In recent years, interest in using EVs as a therapeutic tool has exploded due to their natural properties such as the ability to migrate from one cell to another, carry their contents across the cell membrane, and deliver biologically active cargoes [129]. Recently, PDEVs have been proposed as new nutraceutical and therapeutic tools as they overcome some limitations of M-EVs such as low yield, production cost, toxicity, and immunogenicity [130].

**Figure 2:**
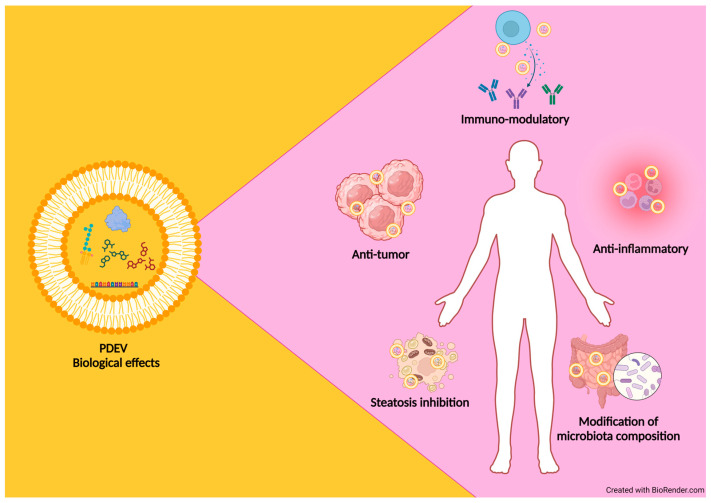
PDEVs as innovative therapeutic tools for treating human diseases.

PDEVs have features that make them natural conveyors of bioactive compounds including terpenes, carotenoids, flavonoids, vitamins, and sugars, which individually or synergically exert beneficial effects on mammalian cells [120,131,132,133]. The main advantages of their possible use as functional carriers are the low toxicity and the significant quantities obtained by the isolation from different parts of edible plants [130]. The sources of PDEVs are multiple and very different from each other; in fact, they have been isolated from several plant species such as ginger, carrot roots [131], grape [109], citrus fruits [134,135,136], ginseng [133], apple [137], wheat [138], garlic [139], and from root exudates of hydroponically grown tomato plants [126]. PDEVs have also been purified from the apoplast of *Arabidopsis thaliana* [125] and *Helianthus annuus* [127]. Even if the molecular content of PDEVs depends on their originating sources, they exert similar biological effects on mammalian cells, i.e., anti-tumor, anti-inflammatory, and immune-modulatory activities (Table 3) [8]. So, as an integral part of the human diet, vesicles derived from fruits and vegetables need to be thoroughly investigated in order to understand their potentially beneficial and/or detrimental effects on humans. It is noteworthy to mention that PDEVs reach the gastrointestinal tract as intact structures, thus participating in the renewal of intestinal tissue and in the modification of intestinal microbiota composition [131]. In addition, PDEVs have been proven to have beneficial functions against inflammatory diseases such as colitis and steatosis [140]. Further, PDEVs are able to inhibit the proliferation of several tumor cell lines, thus representing a new potential approach in cancer treatment [104] (Figure 2).

Raimondo and coworkers [134] demonstrated that PDEVs isolated from citrus lemon juice are able to inhibit the proliferation of three cancer cell lines; the cell proliferation arrest was specific for tumor cells as they did not affect the growth of normal cells. Another study demonstrated that the anti-tumor effect exerted by lemon-derived vesicles in gastric cancer cells could be related to their ability to increase the expression of GADD45A, a tumor suppressor involved in cell cycle control and DNA repair [136].

Growing studies have also demonstrated that PDEVs have anti-inflammatory properties. Consistently, PDEVs isolated from grapes have a protective effect against dextran sulfate sodium (DSS)-induced colitis in mice [109]. This study has demonstrated that grape-derived exosome-like nanoparticles increase the proliferation of Lgr5hi intestinal stem cells both in physiological and pathological conditions. In particular, PDEVs induce the proliferation of intestinal stem cells, thus accelerating the regeneration of mucosal epithelium and restoring the intestinal architecture.

Another study demonstrated that ginger-derived-EVs induced an upregulation of heme oxygenase 1 and interleukin 10 in murine macrophages [131], whose expression in macrophages has been reported to play a crucial role in preventing colitis due to their potent anti-inflammatory activity [141,142]. Similar effects have been observed in RAW 264.7 macrophages treated with ginger-derived EVs. Moreover, PDEVs from grapefruit, ginger, and carrot induce the activation of nuclear factor-like (erythroid-derived 2) [131].

The study of Chen and coworkers [143] demonstrated the capacity of exosome-like nanoparticles isolated from ginger-rhizomes (G-ELNs) to strongly inhibit NLRP3 (nucleotide-binding domain and leucine-rich repeat-containing family, pyrin domain-containing 3) inflammasome activity; this cytoplasmic complex is a key regulator of innate immune responses, and its abnormal activation is linked to the development of various diseases, including Alzheimer’s disease and type 2 diabetes. In particular, lipids associated with G-ELNs are responsible for the inhibitory activity on NLRP3 inflammasome. To obtain this result, total lipids of G-ELNs were extracted, dried, reassembled into liposomes, and then incubated with bone marrow-derived macrophages pretreated with LPS for inflammasome activation. These liposomes substantially suppressed the NLRP3 inflammasome activity, suggesting that the lipids in G-ELNs were the active biomolecules that inhibited the NLRP3 inflammasome activity [143].

In a recent study, Xiao and coworkers [118] evaluated the miRNA expression profile of PDEVs isolated from 11 edible fruits and vegetables (blueberry, coconut, ginger, grapefruit, Hami melon, kiwifruit, orange, pea, pear, soybean, and tomato) by small-RNA sequencing. The target prediction and functional analyses unveiled a significant correlation between the highly expressed miRNAs and pathways associated with the inflammatory response and cancer. These results indicate that miRNAs carried by PDEVs might mediate interspecies intercellular communication.

**Table 3:** Summary of biological properties of PDEVs.

Biological Properties	Source of Pdevs	References
Anticancer	Lemon	[136]
	Citrus limon	[134]
	Grapefruit	[120]
	Ginseng	[133]
	Fruits and vegetables (blueberry, coconut, ginger, grapefruit, Hami melon, kiwifruit, orange, pea, pear, soybean, and tomato)	[118]
Anti-Inflammatory	Broccoli	[140]
	Cabbage	[123]
	Garlic	[139]
	Ginger rhizomes	[143]
	Grape	[109]
	Fruits and vegetables (blueberry, coconut, ginger, grapefruit, Hami melon, kiwifruit, orange, pea, pear, soybean, and tomato)	[118]
Antioxidative	Strawberry	[132]

Compared with synthetic nanoparticles, the biological properties of PDEVs combined with their potential to deliver small-molecule drugs through oral, intravenous, nasal, and transdermal administration make them the best candidate as ‘natural drug delivery tools’.

The advantages linked to the use of PDEVs for therapeutic purposes could be summarized as follows:

- They are natural carriers of biomolecules involved in intercellular communication not only in plants but also in diverse kingdoms of life.
- They are non-toxic and well tolerated by the mammalian immune system as they are currently present in foods.
- Their characteristic lipid membrane composition protects the internal cargo from external agents, which can deteriorate the bioactive compound carried by them.
- Their ability to pass through natural barriers (i.e., blood–brain barrier and the placenta) and reach specific target cells.
- Good yields from vegetable sources; this aspect makes them suitable for industrial applications.
- Possibility of isolating PDEVs at any time, as the source of isolation is represented by numerous plants with different growth periods [144].

In recent years, there has been a great interest in PDEVs; however, knowledge about their cargo, functions, and properties is still in its infancy. Nevertheless, improving our understanding of PDEVs as a holistic entity is of crucial importance for their future use. The acquisition of this knowledge, in fact, could pave the way for the use of PDEVs as nutraceutical and therapeutic tools with the advantage of effectively obtaining them by ecofriendly and cost-effective isolation methods.

## 4. Conclusions and Perspectives

The data summarized in this review highlight that waste and by-products generated from food industries represent not only disposal issues but they are also natural sources of valuable molecules such as proteins, polysaccharides, fibers, flavor compounds, phytochemicals, and nutraceuticals. The isolation of these compounds and their potential applications in bioeconomy and biomedical fields represent one of the main aims of the circular economy.

The revision of literature data indicates that the type and the nature of waste-derived biomolecules depend on the starting agri-food material and the extraction method. The extraction procedures are extremely important for their effects on the yield and should be chosen on the basis of the chemical structures of compounds that have to be isolated. Regarding bioactive molecules with potential biomedical and nutraceutical applications, it can be stated that, independently from the source, the majority of them exhibit antioxidant, anti-proliferative, and antimicrobial activities on mammalian cells. However, some problems have to be solved for their use in the nutraceutical field, starting from the choice of their extraction protocol to their in vivo administration, as several agri-food waste-derived molecules have amphiphilic structures. During purification and storage, these molecules might be degraded or modified. Oxidative and degradative events might also occur during their journey in biological fluids and the intestinal tract. One strategy used to overcome these problems is to encapsulate these molecules in micro and nanostructures, in order to improve their integrity and stability during storage and ensure their active properties for long periods.

In the second part of the review, we shift the focus to the physicochemical and biological properties of EVs as they are natural carriers of bioactive molecules. These molecules, encapsulated within EVs, are protected from degradation and delivered to the target cells. EVs are known to have remarkable potential as next-generation nanocarriers in a wide range of biotechnological and biomedical applications. Since the presence of EVs in foods has been well established, it is reasonable to hypothesize that agri-food wastes may become a valuable source of EVs, reinforcing the concept of waste valorization in the context of the circular economy. In this context, it is important to remark that the procedures currently used for EV isolation are environmentally friendly as they do not require the use of organic solvents. In particular, attention has been paid to the biochemical and biological properties of PDEVs that are characterized by a peculiar protein and lipid content and a cargo composed of a variety of bioactive compounds with beneficial effects on human health, such as anti-inflammatory, anti-tumor, and immune-modulatory activities. Further, PDEVs display some advantages compared with M-EVs such as high yield, low production cost, low toxicity, and antigenicity.

In our opinion, in the future, a promising area of research could be represented by the development of optimized and scalable methods for the isolation of EVs from agri-food wastes, particularly vegetable waste, and the investigation of their biophysical characteristics and biological properties. Recently, EVs have been isolated from olive vegetation water, a by-product generated by olive oil production [9]. The morphology and biochemical composition of these vesicles are similar to those of EVs isolated from edible plants/fruits. Interestingly, EVs isolated from olive vegetation water contain a set of molecules with recognized antioxidant and anti-inflammatory activities [9]. This research field is currently in its infancy; however, agri-food industry wastes might become a sustainable and alternative source of bioactive EVs, which could represent an innovative agri-food by-product to overcome some limitations on the use of the current mammalian and plant sources of EVs.

## Figures and Tables

**Table 1:** Wastes from agriculture and industrial food production.

Agri-Food Industry	Main Waste Origin	Waste Type	Waste Management	References
Beverage industry	Citrus	Pomace and peel	Animal feed	[15]
			Bioactive compound extraction	[17,18,19,20]
	Apple	Pomace	Animal feed; fertilizer	[15]
			Bioactive compound extraction	[21,22,23,26]
	Grape	Stalks and pomace	Soil conditioner and energy	[24]
			Animal feed	[25]
			Bioactive compound extraction	[26,27,28,29]
Olive oil production	Olive	Pomace	Bioactive compound extraction	[30,31,32,33,34]
Tomato industrial processing	Tomato	Peel, seeds, and pulp	Animal feed; fertilizer	[26]
			Bioactive compound extraction	[35,36,37,38]
Dairy industry	Milk	Whey and wastewater	Food derivative preparation	[15]
			Bioactive derivatives	[40,41]
			Biotechnological applications	[11]
Meat processing	Meat	Skin, bones, cartilage, blood, viscera, etc.	Human foods and animal feed	[43,44]
			Biomedical applications	[43,44]
			Technological applications	[42]

**Table 2:** Bioactive compounds from agri-food waste and by-products.

**Bioactive Compounds from Waste of Plant Origin**			
**Bioactive Compound Class**	**Main Waste Source**	**Properties/Bioactivity**	**References**
Pectin	Apple pomace and citrus peel	Gellifier, thickener, and stabilizer	[48]
		Hypocholesterolemia and hypoglycemic effects	[49,50]
		Immunomodulatory and anti-inflammatory	[48,49]
		Antidote against harmful agents	[51]
		Anticancer	[49]
Phenolic compounds	Citrus peel, and apple, grape, and olive pomace	Antioxidant	[46,56,57,58,62,63,64]
		Antimicrobial	[46,56,57,58,62,64]
		Anti-inflammatory	[19,46,62]
		Anticancer	[15,60,61,62]
Anthocyanins	Berry residues, apple peel, and grape pomace	Natural colorants	[69,77]
		Antioxidant and anti-inflammatory	[70,71,72,73]
Natural pigments (betalains, carotenoids, and chlorophylls)	Beetroot pomace, red dragon fruit peel, tomato peel; spinach, lettuce, and broccoli wastes; microalgae residues	Coloring pigments	[69,74]
		Antioxidant, anti-inflammatory, anticancer, antimicrobial, cardio- and neuro-protective activities	[69,77]
**Bioactive Compounds from Waste of Animal Origin**			
**Bioactive Compound Class**	**Main Waste Source**	**Properties/Bioactivity**	**References**
Whey proteins	Whey	Nutritional value	[11,42]
		Anti-hypertensive, antioxidant, anti-obesity, and anticancer	[78]
Meat processing proteins (gelatin, collagen, bioactive peptides, and albumin)	Skin, cartilage, bones, and blood	Emulsifier and gellifier; anti-aging; regenerative activities; nutritional; anti-hypertensive; antioxidant; antithrombotic; antimicrobial	[79,80,81,82,83,84,85]

## Data Availability

No new data were created or analyzed in this study. Data sharing is not applicable to this article.
